# TAK-1/p38/nNFκB signaling inhibits myoblast differentiation by increasing levels of Activin A

**DOI:** 10.1186/2044-5040-2-3

**Published:** 2012-02-07

**Authors:** Anne Ulrike Trendelenburg, Angelika Meyer, Carsten Jacobi, Jerome N Feige, David J Glass

**Affiliations:** 1 Novartis Institutes for Biomedical Research, Forum 1, Novartis Campus, 4056 Basel, Switzerland; 2 Novartis Institutes for Biomedical Research, 100 Technology Square, Cambridge, MA 02139, USA

## Abstract

**Background:**

Skeletal-muscle differentiation is required for the regeneration of myofibers after injury. The differentiation capacity of satellite cells is impaired in settings of old age, which is at least one factor in the onset of sarcopenia, the age-related loss of skeletal-muscle mass and major cause of frailty. One important cause of impaired regeneration is increased levels of transforming growth factor (TGF)-β accompanied by reduced Notch signaling. Pro-inflammatory cytokines are also upregulated in aging, which led us hypothesize that they might potentially contribute to impaired regeneration in sarcopenia. Thus, in this study, we further analyzed the muscle differentiation-inhibition pathway mediated by pro-inflammatory cytokines in human skeletal muscle cells (HuSKMCs).

**Methods:**

We studied the modulation of HuSKMC differentiation by the pro-inflammatory cytokines interleukin (IL)-1α and tumor necrosis factor (TNF)-α The grade of differentiation was determined by either imaging (fusion index) or creatine kinase (CK) activity, a marker of muscle differentiation. Secretion of TGF-β proteins during differentiation was assessed by using a TGF-β-responsive reporter-gene assay and further identified by means of pharmacological and genetic inhibitors. In addition, signaling events were monitored by western blotting and reverse transcription PCR, both in HuSKMC cultures and in samples from a rat sarcopenia study.

**Results:**

The pro-inflammatory cytokines IL-1α and TNF-α block differentiation of human myoblasts into myotubes. This anti-differentiation effect requires activation of TGF-β-activated kinase (TAK)-1. Using pharmacological and genetic inhibitors, the TAK-1 pathway could be traced to p38 and NFκB. Surprisingly, the anti-differentiation effect of the cytokines required the transcriptional upregulation of Activin A, which in turn acted through its established signaling pathway: ActRII/ALK/SMAD. Inhibition of Activin A signaling was able to rescue human myoblasts treated with IL-1β or TNF-α, resulting in normal differentiation into myotubes. Studies in aged rats as a model of sarcopenia confirmed that this pro-inflammatory cytokine pathway identified is activated during aging.

**Conclusions:**

In this study, we found an unexpected connection between cytokine and Activin signaling, revealing a new mechanism by which cytokines affect skeletal muscle, and establishing the physiologic relevance of this pathway in the impaired regeneration seen in sarcopenia.

## Background

Regeneration of myofibers after injury requires skeletal-muscle differentiation [1,2]. It has been shown in studies of old age that the differentiation capacity of satellite cells is impaired, which is at least one factor in the onset of sarcopenia, the age-related loss of skeletal-muscle mass and strength [3]. Myoblast differentiation is influenced by a number of factors. For example, insulin-like growth factor (IGF)-1 and low serum conditions promote differentiation [4-6], whereas transforming growth factor (TGF)-β and its family members, such as myostatin, block differentiation [7-13] as do pro-inflammatory cytokines [14-16].

The role of the pro-inflammatory cytokines, particularly in skeletal-muscle differentiation, is controversial, as there are conflicting reports, documenting the capacity of these cytokines to either induce [17-19] or inhibit [14-16,19] differentiation. Tumor necrosis factor (TNF)-α was found to be required for myogenesis, as shown by impaired regeneration in TNF-α null animals [20]; however, the concentration of TNF-α required to promote differentiation is apparently very low, and higher levels can have the opposite effect; for example, whereas 0.05 ng/ml of TNF-α stimulated myogenesis, 0.5 and 5 ng/ml caused inhibition [19]. Similarly, the role of the downstream p38 pathway is under some dispute. On the one hand, the activity of p38 mitogen-activated protein kinase (MAPK) is reportedly increased during myogenesis, and its inhibition was shown to inhibit the expression of select muscle-specific genes and formation of multinucleated myotubes [21]. During myogenesis, the activation of p38 MAPK promotes cell cycle exit by inducing the expression of a cyclin-dependent kinase inhibitor, p21, which facilitates terminal differentiation of muscle precursor cells [22]. On the other hand, however, there are multiple reports of p38 inhibiting myogenesis; for example, MAPK kinase kinase (MEKK)1 signaling through p38 was shown to result in the inactivation of E47 and thus repress myogenesis [23], and p38 phosphorylation of the transactivation domain of myogenic regulatory factor (MRF)4 represses transcription of myogenic genes [24].

The phosphoinositide 3-kinase (PI3K)/AKT pathway is also activated during myogenesis, and insulin-like growth factor (IGF)-1, which initiates PI3K/AKT signaling, is able to induce both differentiation of myoblasts [5,6], and hypertrophy of post-differentiated myotubes [25-29]. In post-differentiated muscle, IGF-1/PI3K/AKT signaling opposes the action of TNF-α/NFκB activity, for example by inhibiting NFκB-mediated upregulation of the E3 ubiquitin ligases MuRF1 and MAFbx, which are required for skeletal muscle atrophy [30].

TGF-β-activated kinase 1 (TAK-1), a member of the MEKK (MAP3K) family, was identified as a regulator of TGF-β-induced activation of MAPK [31]. Recent studies have shown that TAK-1 is also a component of signaling pathways leading to the activation of NFκB and activator protein-1 in response to diverse cytokines, including interleukin (IL)-1 and TNF-α [32-37]. However, the role of TAK-1 in muscle progenitor cells has not been definitively determined, although a recent study claimed that TAK-1 is essential for the differentiation of myoblasts, and is required for the myogenic actions of IGF-1 [38]. This was unexpected, because TGF-β molecules themselves have been shown in multiple studies to block muscle differentiation [13,39-41], suggesting that TAK-1 is a negative modulator of muscle differentiation. In the present study, we found that TAK-1 connects TNF-α and IL-1 to Activin signaling, explaining how these cytokines can inhibit myogenesis.

## Methods

### Cell culture and treatment

Human skeletal muscle cells (HuSKMCs; Lonza AG, Basel, Switzerland) were cultured in growth medium (GM) consisting of skeletal muscle basal medium (skBM; Lonza) supplemented with 20% FCS (PAA Laboratories, Dartmouth, PA, USA). Differentiation was initiated 24 to 48 hours after seeding by changing to a serum-free differentiation medium (DM), skBM. For small interfering (si)RNA experiments, cells were transfected 24 hours after seeding in GM, and differentiation was initiated after another 24 hours.

To determine NFκB activity, HuSKMCs were infected 24 hours after seeding with human recombinant adenovirus-NFκB-luciferase (Vector Laboratories Inc., Burlingame, CA, USA) in GM for 48 hours, then the medium was removed and the cells stimulated for another 6 hours in serum-free skBM with the compounds under investigation To assess the effects on HuSKMC differentiation, the assessed compounds were added at the onset of differentiation, and cells were differentiated into myotubes for up to 120 hours. To measure TGF-β reporter-gene activity in supernatants (SNs) from differentiating HuSKMCs, a reporter-gene assay (RGA) was used. HEK293T cells stably transfected with pGL3-CAGA12-luc were seeded in serum-reduced medium (2% FCS) for 24 hours, then the medium was removed, and the cells stimulated with a 10:1 mixture of supernatant and serum-enriched medium (20% FCS) in the absence or presence of 500 ng/ml of a human Fc-TGF-β RIIb/Fc chimera (TGF-βRIIb) alone or in combination with neutralizing anti-Activin A antibody (αActA) for another 24 hours.

### Biochemistry

The following reagents were used: human IL-1α), IL-1β, TNF-α, TGF-βRIIb, andαActA (all R&D Systems Inc., Minneapolis, MN, USA), long-R3-iIGF-1, SB431542 (both Sigma-Aldrich, St Louis, MO, USA), SB203580, withaferin A (both Tocris Bioscience, Ellsville, MO, USA), and TAK-1 inhibitor (AnalytiCon Discovery AG, Potsdam, Germany). Stock solutions were prepared either in PBS supplemented with 0.1% BSA for Fc-TGF-βRIIBb, TNF-α and IL-1α, in 10 mmol/l HCl for IGF-1 or in dimethyl sulfoxide (DMSO) for TAK-1 inhibitor, SB431542, SB203580, and withaferin A. For immunostaining, a primary antibody against myosin heavy chain (MyHC) was used (clone A4.1025; Upstate Biotechnology, Lake Placid, NY, USA), and the secondary antibody was conjugated to a fluorescent dye (Alexa Fluor^® ^488 F (AB'); Invitrogen Corp., Carlsbad, CA, USA).

Primary antibodies against phospho-TAK-1 (Thr 184/187), phospho-SEK/MKK4 (Thr241), phospho-p38MAPK (Thr 180/Tyr182), phospho-c-Jun (Ser63), phospho-activating transcription factor (ATF)2 (Thr71), phospho-NFκB p65 (Ser536), phospho-SMAD2 (Ser465/467), phospho-AKT (Ser473) (all Cell Signaling Technology, Beverly, MA, USA), and phospho-SMAD3 (Ser423/425; Millipore Corp., Billerica, MA, USA) were used for western blotting. The loading control was α-tubulin (Sigma), and the secondary antibodies were labelled with horseradish peroxidase (HRP) (goat anti-rabbit IgG HRP and goat anti-mouse IgG HRP; Cell Signaling Technology).

### Western blotting

Lysis buffer consisting of extraction reagent (Phosphosafe; Novagen Inc., Madison, WI, USA) supplemented with 1% protease inhibitor cocktail (Sigma-Aldrich) was added. Homogenates were separated by centrifugation for 10 minutes at 4°C (14,000 rpm). Supernatants were collected and protein contents measured a commercial kit for protein determination (BCA Kit; Sigma-Aldrich). Samples were diluted in SDS-PAGE sample buffer and denatured for 5 minutes at 95°C. Equal amounts of protein were loaded per lane of 4 to 12% polyacrylamide gel (NuPAGE Bis-Tris gel; Invitrogen Corp., Carlsbad, CA, USA), separated by electrophoresis, and then transferred onto nitrocellulose membranes. Membranes were blocked in TBS with 5% w/v non-fat milk powder. Primary antibodies were incubated in TBS with 0.1% Tween 20 and 5% BSA with the exception of phospho-SMAD2 (incubated with 5% non-fat milk), and secondary antibodies in TBS with 0.1% Tween 20, 0.05% SDS and 5% non-fat milk. Immunoreactivity was detected by SuperSignal West Femto Maximum Sensitivity Substrate and exposed to film.

### RNA analysis

RNA was isolated using commercial kits (RNEasy Mini Kit, RNEasy MinElute Kit and RNase-Free DNase Set; Qiagen Inc., Valencia, CA, USA), following the manufacturer's protocol. Samples were prepared for reverse transcription PCR (QuantiFAST Multiplex RT-PCR+R Kit; Qiagen Inc.) and run in an automated PCR machine (7500 FAST Real-time PCR machine; Applied Biosystems, Foster City, CA, USA). A TaqMan probe set for the Activin A β-chain was used (Inhibin β A; Rn00567500_m1: rat; Hs01081598_m1: human; Applied Biosystems, Foster City, CA, USA).

### Small interfering RNA

The siRNA pools used were designed against SMAD2, SMAD3, the Activin A β-chain (Inhibin β A), and a non-targeting control (NTC) targeting an unknown mammalian sequence together with transfection reagent (DharmaFECT 1; Dharmacon/Thermo Fisher Scientific Inc., Rockford, IL, USA) were used, to give a final siRNA concentration of 100 nmol/L.

### Immunostaining

After washing with cytoskeleton stabilizing buffer (CSB; 80 mmol/l PIPES, 5 mmol/l EGTA, 1 mmol/l MgCl_2_, 40 g/l PEG3500 in distilled water at pH7.4), cells were fixed with 4% paraformaldehyde in CSB. Cells were then permeabilized with 0.2% Triton in CSB, and non-specific binding blocked with normal goat serum (Zymed Laboratories Inc., South San Francisco, CA, USA) followed by incubation with MyHC diluted in PBS and subsequently with fluorescent dye (Alexa Fluor^® ^488 F (AB'); Invitrogen Corp.) diluted in PBS. Cells wre mounted with in an antifade reagent with DAPI (ProLong Gold; Invitrogen Corp.).

### Reporter-gene assays

HEK293T cells stably transfected with the TGF-β responsive construct CAGA12-luc cloned into the reporter construct pGL3 (Promega Corp., Madison, WI, USA) were kindly provided by C. Lu (Developmental and Molecular Pathways, Novartis Pharma AG, Basel, Switzerland). Infection of HuSKMCs was performed using the human recombinant adenovirus-NFκB-luciferase reporter (Vector Laboratories Inc., Burlingame, CA, USA). Reporter-gene activity was measured using Britelite Plus (Perkin Elmer, Waltham, MA, USA), and chemiluminescence was read using a spectrophotometer (Spectramax M5; Molecular Devices Inc., Sunnyvale, CA, USA).

### Creatine kinase activity assay

Cells were washed three times with PBS and then lysed with reporter lysis buffer (Promega Corp.) and stored at -80°C until measurement. Creatinine kinase (CK) activity was measured using a commercial reagent (CK (IFCC) Reagent; Thermo Electron, Waltham, MA, USA), prepared according to the manufacturer's instructions. Lysates were adjusted to room temperature, CK reagent was added, and absorbance was immediately read at 340 nm for 20 minutes with a reading interval of 1 minute. CK standard curves were freshly prepared using CK from rabbit muscle (Roche Diagnostics, Basel, Switzerland). Protein content was determined using a commercial (BCA Kit; Sigma-Aldrich), as before.

### Activin A ELISA

ELISA for Activin A (Human Activin A DuoSet; R&D Systems Inc.) was performed according to the manufacturer's instructions, using a modified chemiluminescent measurement. Briefly, plates were coated with capture antibodies overnight at 4°C, and non-specific binding was blocked with PBS plus 1% BSA for at least 1 hour. Supernatants and 6M Urea (1:1) were added and incubated for 2 hours at room temperature. Detection antibody was added for 2 hours at room temperature followed by incubation with HRP-labeled streptavidin for 20 minutes at room temperature. After addition of a chemiluminescent substrate (SuperSignal ELISA Femto Maximum Sensitivity substrate (PierceBiotechnology Inc., Rockford, Illinois, USA) chemiluminescence was read using a spectrophotometer (Spectramax M5; Molecular Devices Inc.).

### *In vivo *experiments

All animal procedures were approved by the cantonal veterinarian office of Basel (license number 2127).

Male Wistar rats (Janvier, Le Genest Saint Isle, France) of various ages (4 per group) were housed for 2 weeks on a 12 hour light/dark cycle with unrestricted access to food and water. Rats were asphyxiated using CO_2 _at 6, 18, 21 and 24 months (29, 81, 93 and 106 weeks) of age, and the gastrocnemius muscle was immediately dissected, weighed and snap-frozen in liquid nitrogen before processing for RNA extraction. Compared with 6-month-old rats, the gastrocnemius weight was unchanged for 18-month-old rats and reduced by 41% and 49% in 21- and 24-month-old rats, respectively.

### Statistical analysis

Differences between groups were analyzed using one-way ANOVA for all experiments. *P *< 0.05 was considered significant.

## Results and discussion

### The transforming growth factor-β/SMAD and insulin-like growth factor-1/AKT pathways modulate interleukin-1α- and tumor necrosis factor-α-induced inhibition of human skeletal myoblast differentiation

**Figure 1:**
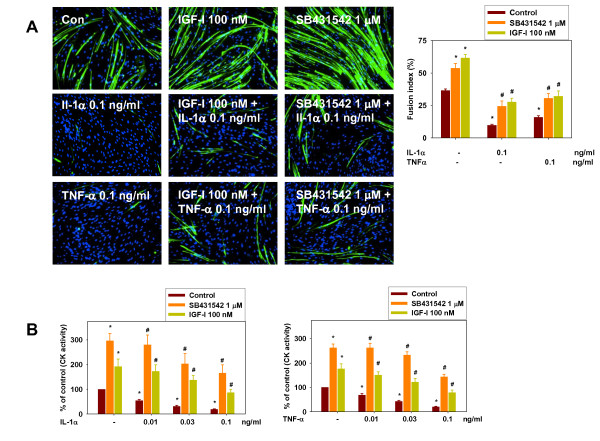
**Insulin-like growth factor (IGF)-1 and ALK inhibition counteracts interleukin (IL)-1α- and tumor necrosis factor (TNF)-α-induced inhibition of human skeletal muscle cell (HuSKMC) differentiation**. **(A) **HuSKMC myotubes differentiated for 5 days in the absence (Con) and presence of IL-1α (0.1 ng/ml) and TNF-α (0.1 ng/ml), alone and in combination with IGF-1 (100 nmol/l) or SB431542 (1 μmol/l), were stained with anti-myosin heavy chain (MyHC) and DAPI. Shown are representative pictures and analysis of fusion index which was determined as the percentage of nuclei occurring in myotubes stained with MyHC on four pictures. Data are means ± SEM from three to six independent experiments. Differences from untreated HuSKMCs (control; first column),*P < 0.05; differences from IL-1α- and TNF-α-treated HuSKMCs (control; second and third column), #P < 0.05. **(B) **Analysis of creatine kinase (CK) activity in HuSKMC myotubes differentiated for 4 days and treated with either IL-1α (0.01-0.1 ng/ml) and TNF-α, alone (0.01 to 0.1 ng/ml) or in combination with IGF-1 (100 nmol/l) or SB431542 (1 μmol/l). Data are expressed as percentage of control untreated HuSKMCs. Data are means ± SEM from four to nine independent experiments. Differences from untreated HuSKMCs (control; first column),*P < 0.05; differences from IL-1α- and TNF-α-treated HuSKMCs (control; second to fourth columns), #P < 0.05.

In this study, we were interested in determining the effect of cytokines on human skeletal myoblast differentiation. As a first step, it was important to characterize whether HuSKMCs [13] respond to cytokines in a similar manner to myoblast lines from other species. We used IL-1α to stimulate IL-1 receptors throughout this study, but very similar effects were seen whenever IL-1β was tested as well (data not shown). HuSKMCs were differentiated in the absence or presence of IL-1α and TNF-α for 5 days after the onset of differentiation, and immunostained with antibodies to MyHC as a marker for differentiation (Figure 1A, left panel). Similar to previous studies, both IL-1α and TNF-α caused a marked reduction in HuSKMC differentiation, seen as a decrease in both myotube number (Figure 1A, left panel) and fusion index (FI), which were decreased by 73% with IL-1α and by 55% witu TNF-α (Figure 1A, right panel). Moreover, CK activity, which normally increases during differentiation, was also markedly reduced by IL-1α and TNF-α (Figure 1B); no increase in CK activity was seen at any concentration tested (0.001 to 1 ng/ml; data not shown).

We next investigated whether the anti-differentiation effect of IL-1α and TNF-α on HuSKMCs is perturbed by IGF-1, a positive regulator of myogenesis. Treatment with IGF-1 promoted basal differentiation of HuSKMCs by up to 77%, and partially rescued them from the inhibitory effects of IL-1α and TNF-α, as determined by FI (Figure 1A, right panel) and CK activity (Figure 1B). Because IGF-1-mediated AKT signaling has been shown to block the inhibition of differentiation caused by TGF-β family members [13], we investigated whether the intersection between cytokine signaling and IGF-1 signaling might involve the TGF-β pathway. Therefore we sought to determine the influence of the TGF-β/ALK pathway in IL-1α and TNF-α action, using the ALK4/5/7 inhibitor SB431542. Treatment with SB431542 promoted basal HuSKMC differentiation by up to threefold and partially rescued the blocking of differentiation caused by IL-1α and TNF-α, analyzed by either FI (Figure 1A, right panel) or CK activity (Figure 1B) suggesting that TGF-β/ALK signaling may play a role in the IL-1α and TNF-α-induced inhibition of HuSKMC differentiation.

### Interleukin-1α and tumor necrosis factor-α induce secretion of Activin A via activation of the transforming growth factor-β-activated kinase (TAK)-1/p38/nuclear factor κB pathway during human skeletal muscle cells differentiation; secretion is independent of SMAD2/3

The fact that the anti-myogenesis effects of IL-1α and TNF-α could be blocked using an ALK inhibitor suggested either that these two pathways were acting in parallel, and that the ALK inhibitor simply perturbed the basal tone of differentiation, or that there could be an increase in activation of the TGF-β receptor/ALK pathway upon cytokine treatment. This might occur via an increase in the production and subsequent secretion of TGF-β family-member proteins. We therefore sought to determine if TGF-β protein secretion from differentiating HuSKMCs contributes to the IL-1α and TNF-α inhibition of myogenesis. Supernatants from HuSKMCs differentiated in the absence and presence of IL-1α and TNF-α were analyzed in an RGA, using as activity marker CAGA-luc (a luciferase reporter for SMAD2/3 transcription factor), which is sensitive to most TGF-β proteins including the TGF-β isoforms TGF-β1, TGF-β2 and TGF-β3, the activins, myostatin, and growth differentiation factor (GDF)-11.

**Figure 2:**
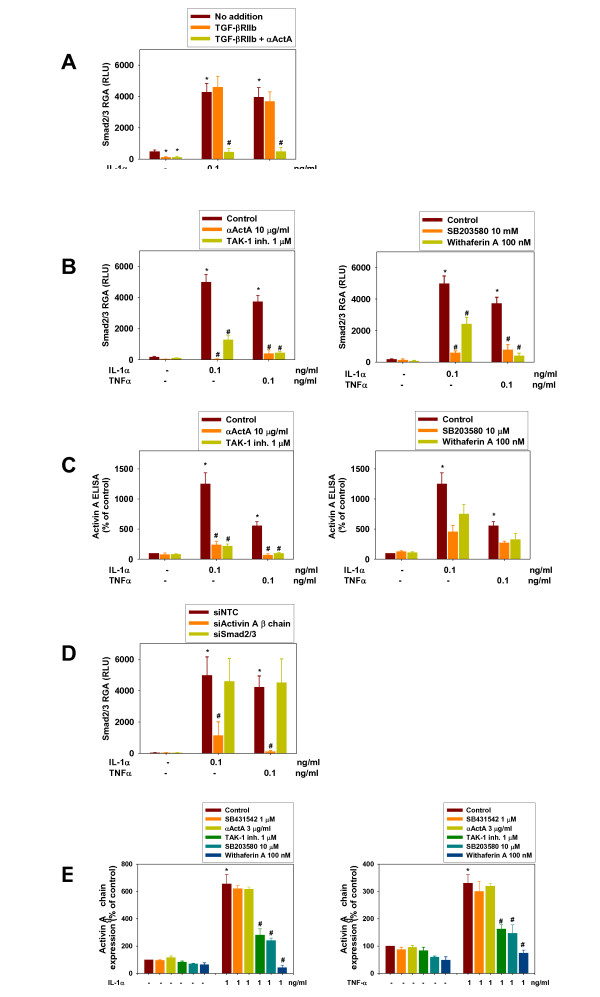
**Interleukin (IL)-1**α **and tumor necrosis factor (TNF)-**α **induced secretion of Activin A via activation of transforming growth factor-β-activated kinase (TAK)-1, p38 and nuclear factor (NF)κB pathways during human skeletal muscle cell (HuSKMC) differentiation**. Supernatants (SNs) from HuSKMC myotubes differentiated for 3 days were analyzed in **(A,B,D) **HEK293Tcells stably transfected with the SMAD-sensitive CAGA-luc reporter in a reporter-gene assay (RGA) to assess occurrence of active TGF-β proteins or **(C) **with Activin A ELISA. **(E) **Activin A β-chain mRNA in HuSKMC myotubes differentiated for 6 hours was analyzed by reverse transcriptase (RT)-PCR. HuSKMCs were differentiated in the absence or presence of IL-1α (0.1 ng/ml) or TNF-α (0.1 ng/ml), alone or in combination **(A-C) **with the neutralizing anti-Activin A antibody (αActA), TAK-1 inhibitor, the p38 inhibitor SB203580 or the NFκB inhibitor withaferin A, or **(D) **after small interfering (si)RNA treatment. Data are expressed as **(A,B,D) **relative light units (RLU) or as percentage of control untreated HuSKMCs for **(C) **Activin A ELISA and **(E) **RT-PCR. Data are means ± SEM from 3 to 17 independent experiments. Differences from untreated HuSKMCs,*P < 0.05; differences from IL-1α- and TNF-α-treated HuSKMCs, #P < 0.05. **(A) **SNs were analyzed in CAGA-luc RGA. The soluble TGF-βRIIb/Fc chimera (500 ng/ml; TGF-βRIIb) was added alone to the supernatant to determine the contribution of the TGF-β isoforms (TGF-β1, TGF-β2 and TGF-β3) to CAGA-luc activity or in combination with αActA (10 μg/ml; TGF-βRIIb + αActA) to assess contribution of Activin A. **(B) **SNs were analyzed in CAGA-luc RGA. To eliminate responses to the TGF-β isoforms, CAGA-luc activity was measured after addition of TGF-βRIIb to the SN. **(C) **SNs were analyzed with Acitivin A ELISA. **(D) **SNs were analyzed in CAGA-luc RGA after addition of TGFβRIIb. SN were taken after treatment with siRNA against non-targeting control (siNTC), Activin A β-chain (siActivin A β-chain) or SMAD2 and SMAD3 (siSMAD2/3). **(E) **Activin A β-chain mRNA were analyzed by RT-PCR. Inhibitors were given 3 hours before IL-1α or TNF-α stimulation.

Supernatants from untreated HuSKMCs induced a small degree of SMAD2/3 CAGA-luc activity (Figure 2A), confirming autocrine secretion of active TGF-β proteins from differentiating HuSKMCs.

We next determined which TGF-β family-member proteins are secreted from HuSKMCs by adding pharmacologic inhibitors to the supernatant. In supernatant from untreated HuSKMCs, SMAD2/3 activity mainly represents TGF-β isoforms, as indicated by the marked reduction of SMAD2/3 CAGA-luc activity (by about 74%) after the soluble TGF-βRIIb/Fc chimera (Figure 2A, TGF-βRIIb) was added to the supernatant, and the lack of further reduction after addition of either a neutralizing Activin A antibody (Figure 2A, αActA) or follistatin (a natural blocker of myostatin; data not shown), GDF-11, and activins. Supernatants harvested from HuSKMCs showed markedly increased CAGA-luc activity after treatment with IL-1α and TNF-α, with increases of 776% and 711%, respectively *Figure 2A). Addition of TGF-βRIIb to the supernatant did not change SMAD2/3 activity (Figure 2A, TGF-βRIIb), whereas αActA (Figure 2A, αActA) almost completely abolished SMAD2/3 activity, indicating that IL-1α and TNF-α specifically result in secretion of Activin A from differentiating HuSKMCs.

To directly measure the levels of Activin A protein produced by stimulating IL-1α and TNF-α, an Activin ELISA was used, which showed that Activin A levels were increased by 1,152% and 459% after treatment with IL-1α and TNF-α, respectively (Figure 2C).

To determine the signaling pathways responsible for IL-1α- and TNF-α-induced Activin A secretion from differentiating HuSKMCs, the SMAD2/3-induced luciferase activity was analyzed, using supernatants harvested from HuSKMCs treated with IL-1α and TNF-α, either alone or in combination with pathway inhibitors shown to modulate IL-1α and TNF-α effects. CAGA-luc activity induced by IL-1α and TNF-α was significantly reduced by a TAK-1 inhibitor (Figure 2B), as was Activin A level (Figure 2C), establishing a good correlation between the two parameters. The same correlation was seen with a p38 inhibitor, SB203580, which produced a decline in both SMAD2/3 activation (Figure 2B) and Activin A levels (Figure 2C), and an NFκB inhibitor, withaferin A, (Figure 2B,C), whereas the Jun kinase (JNK) inhibitor SP600125 did not cause any changes (data not shown). These data suggest that the IL-1α- and TNF-α-induced secretion of Activin A requires TAK-1/p38/NFκB signaling, but seem to be independent of JNK.

Genetic approaches were also used to determine the requirement for IL-1α- and TNF-α-induced Activin A expression to produce the resulting SMAD2/3 signaling. HuSKMCs were treated before differentiation with siRNAs directed against either the Activin A β chain or (as an experiment to determine where SMAD2/3 signaling comes into play) to SMAD2 and SMAD3 (siSMAD2/3), and then differentiated in the absence or presence of IL-1α and TNF-α. SMAD2/3 CAGA-luc activity was analyzed after treatment with the resulting supernatant. The siActivin A β-chain almost completely abolished SMAD2/3 CAGA-luc responses (Figure 2D) induced by IL-1α and TNF-α treatment of HuSKMCs, suggesting that the observed increase in Activin A secretion is dependent on *de novo *synthesis. By contrast, siSMAD2/3 inhibition of the SMAD pathway in the IL-1α-or TNF-α-treated HuSKMCs did not alter the SMAD2/3 CAGA-luc activity of the supernatant (Figure 2D), indicating that activation of the ALK/SMAD2/3 pathway is downstream of Activin A secretion. To determine the requirement for IL-1α and TNF-α pathway stimulation for Activin A secretion, expression of Activin A β chain was analyzed by RT-PCR in HuSKMCs treated for 6 hours with IL-1α and TNF-α, either alone or in combination with various pathway inhibitors. Both IL-1α and TNF-α alone increased expression of Activin A β-chain (Figure 2E). These effects were not influenced by SB431542 or αActA, but were markedly reduced by SB203580, withaferin A, and TAK-1 inhibitor (Figure 2E), confirming that IL-1α and TNF-α induce Activin A *de novo *synthesis via TAK-1/p38/NFκB signaling. Experiments with IL-1β (data not shown) in HuSKMCs confirmed activation of this TAK-1/p38/NFκB pathway by IL-1β as well.

### Transforming growth factor-β-activated kinase-1/p38/nuclear factorκB-dependent Activin A secretion mediates interleukin-1α and tumor necrosis factor-α-induced inhibition of human skeletal muscle cell differentiation, which requires SMAD2/3

**Figure 3:**
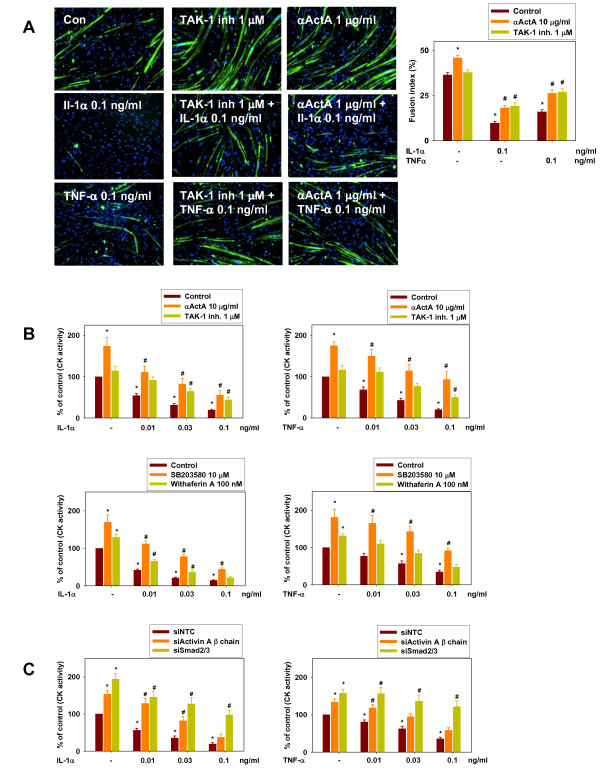
**Inhibition of human skeletal muscle cell (HuSKMC) differentiation by interleukin (IL)-1**α **and tumor necrosis factor (TNF)-**α **is mediated by transforming growth factor-β-activated kinase (TAK)-1/p38/nuclear factor (NF)κB/Activin A/SMAD2/3 pathway**. **(A) **HuSKMC myotubes differentiated for 5 days in the absence (Con) and presence of IL-1α (0.1 ng/ml) or TNF-α (0.1 ng/ml), alone and in combination with the neutralizing neutralizing anti-Activin A antibody (10 μg/ml; αActA) or TAK-1 inhibitor (1 μmol/l) were stained with anti-myosin heavy chain (MyHC) and DAPI. Shown are representative pictures and analysis of fusion index, which was determined as the percentage of nuclei occurring in myotubes stained with MyHC on four pictures taken. Data are means ± SEM from four to six independent experiments. Differences from untreated HuSKMCs (control; first column),*P < 0.05; differences from IL-1α- and TNF-α-treated HuSKMCs (control, second and third column), #P < 0.05. **(B) **Analysis of CK activity from in HuSKMC myotubes differentiated for 4 days and treated with either IL-1α (0.01-0.1 ng/ml) and TNF-α alone (0.01-0.1 ng/ml), and in combination with αActA (10 μg/ml), TAK-1 inhibitor (1 μmol/l), SB203580 (10 μmol/l) or withaferin A (100 nmol/l). Data are expressed as percentage of control from untreated HuSKMCs. Data are means ± SEM from three to nine independent experiments. Differences from untreated HuSKMCs (control; first column),*P < 0.05; differences from IL-1α- and TNF-α-treated HuSKMCs (control, second to fourth columns), #P < 0.05. **(C) **Analysis of CK activity from in HuSKMC myotubes differentiated for 4 days and treated with IL-1α (0.01-0.1 ng/ml) and TNF-α alone (0.01-0.1 ng/ml) after treatment with either small interfering (si)RNA against non-targeting control (siNTC), siActivin A β-chain or siSMAD2/3. Data are means ± SEM from five to seven independent experiments. Differences from siNTC-treated HuSKMCs (siNTC; first column),*P < 0.05; differences from IL-1α- and TNF-α-treated HuSKMCs (siNTC, second to fourth columns), #P < 0.05.

We next assessed whether Activin A secretion induced by IL-1α and TNF-α contributes to the inhibitory effect of these cytokines on HuSKMC differentiation. αActA and TAK-1 (p38 and NFκB inhibitors) were tested in the presence of IL-1α and TNF-α. HuSKMCs were differentiated in the absence or presence of IL-1α and TNF-α, alone or in combination with αActA or inhibitors. Again, IL-1α and TNF-α alone caused a marked reduction in HuSKMC differentiation, as determined by myotube number (Figure 3A, left panel), FI (Figure 3A, right panel) and CK activity (Figure 3B,C). αActA promoted basal HuSKMC differentiation (by up to 80%) and partially rescued it from the inhibitory effects of IL-1α and TNF-α as determined either by FI (Figure 3A, right panel) or CK activity (Figure 3B) showing that inhibition of differentiation by IL-1α and TNF-α requires Activin A secretion. TAK-1 inhibitor (Figure 3A, right panel; Figure 3B), p38, SB203580 (Figure 3B) and the NFκB inhibitor withaferin A (Figure 3B) all tended to rescue differentiation from the inhibitory effects of IL-1α and TNF-α, confirming the requirement of TAK-1/p38/NFκB signaling on blockade of differentiation. In fact, SB203580 and withaferin A increased basal CK activity by up to 81% and 32%, respectively (Figure 3B) confirming the contribution of endogenous p38/NFκB signaling to the basal tone of HuSKMC differentiation.

Genetic inhibition with siActivin A β-chain and siSMAD2/3 treatment also increased CK activity, by up to 54% and 94%, respectively, and rescued it from rescued it from the inhibitory effects of IL-1α and TNF-α (Figure 3C). These data confirm the dependence of IL-1α- and TNF-α-mediated inhibition of differentiation on the induction of Activin A *de novo *secretion and subsequent activation of ALK/SMAD signaling.

### Interleukin-1α and tumor necrosis factor-α signal via transforming growth factor-β-activated kinase-1 p38/nuclear factor κB and subsequently Activin A/SMAD2/3/AKT in differentiating human skeletal muscle cells

**Figure 4:**
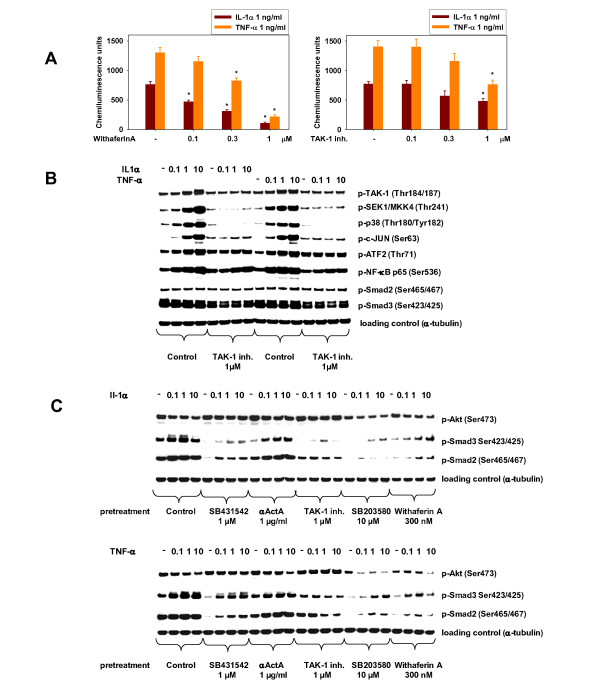
**Inhibition of human skeletal muscle cell (HuSKMC) differentiation by interleukin (IL)-1**α **and tumor necrosis factor (TNF)-**α **is mediated by the transforming growth factor-β-activated kinase (TAK)-1/p38/nuclear factor (NF)κB/Activin A/SMAD2/3 pathway**. **(A) **Analysis of NFκB-luc assay (RGA) from HuSKMC myoblasts treated with IL-1α (1 ng/ml) and TNF-α (1 ng/ml) alone, and in combination with TAK-1 inhibitor (0.1-1 μmol/l) or withaferin A (0.1-1 μmol/l). Data are expressed as relative light units (RLU). Data are means ± SEM from four independent experiments. Differences from IL-1α- and TNF-α-treated HuSKMCs (first column),*P < 0.05. **(B) **Immunoblotting of phospho-TAK-1, phospho-SEK1/MKK4, phospho-p38MAPK, phospho-c-Jun, phospho-ATF2, phospho-NFκB, phospho-SMAD2 and phospho-SMAD3 of samples from HuSKMCs treated with TNF-α (0.1 to 10 ng/ml) or IL-1α (0.1 to 10 ng/ml) for 15 minutes alone, and in the presence of TAK-1 inhibitor (1 μmol/l) starting at the onset of differentiation. The TAK-1 inhibitor was given 3 hours before IL-1α or TNF-α stimulation. Shown are representative immunoblots. **(C) **Immunoblotting of phospho-SMAD2, phospho-SMAD3 and pAKT of samples from HuSKMCs treated with TNF-α (0.1 to 10 ng/ml) or IL-1α (0.1 to 10 ng/ml) for 24 hours, alone and in the presence of SB431542 (1 μmol/l), αActA (10 μg/ml), TAK-1 inhibitor (1 μmol/l), SB203580 (10 μmol/l) or withaferin A (300 nmol/l) starting at the onset of differentiation. Inhibitors were given 3 hours before IL-1α or TNF-α stimulation. Shown are representative immunoblots.

Signaling experiments were performed in differentiating HuSKMCs, using either analysis of NFκB activity (Figure 4A) or western blotting (Figure 4B,C) to determine the contributing pathways required for Activin A release. NFκB signaling was assessed by an adenoviral NFκb-Luciferase reporter. The NFκB CAGA-luc activity induced by IL-1α and TNF-α was counteracted by TAK-1 inhibitor and by withaferin A (Figure 4A) indicating that TAK-1 is involved in IL-1α and TNF-α activation of NFκB signaling and, thus is upstream of NFκB. However, TAK-1 inhibitor was less efficacious than withaferin in blocking NFκB signaling, indicating only partial NFκB inhibition by TAK-1.

We next analyzed HuSKMCs stimulated with IL-1α and TNF-α, either alone or in combination with TAK-1 inhibitor, using phospho-specific antibodies for signaling molecules (Figure 4B). Both IL-1α and TNF-α (and IL-1β; data not shown) increased phosphorylation of TAK-1, MKK4, p38, c-Jun, ATF2, NFκB, and p65 in a concentration-dependent manner (Figure 4B). TAK-1 inhibitor markedly reduced phosphorylation by IL-1α and TNF-α (Figure 4B), indicating that TAK-1 is upstream of NFκB, MKK, p38, c-Jun, and ATF2. By contrast, SMAD2/3 phosphorylation remained unchanged by this short treatment with IL-1α and TNF-α (Figure 4B), in agreement with the observation that immediate Activin A secretion is independent of SMAD2/3, but secreted Activin A subsequently signals through SMAD2/3.

To further test this model, HuSKMCs stimulated for 24 hours with IL-1α and TNF-α, alone or in combination with various inhibitors, were analyzed. Secreted activin A after 24 hours of treatment was assessed by measuring TGF-β CAGA-luc activity from supernatants (data not shown). At this later time point, both IL-1α and TNF-α resulted in an increase in phosphorylation of SMAD3, but SMAD2 was increased only marginally, and only upon IL-1α stimulation (Figure 4C). In addition, there was a decrease in phosphorylation of AKT (Figure 4C) which has been previously shown to be a consequence of ALK/SMAD(2)/3 signaling in skeletal muscle cells (Figure 4C). Therefore, AKT inhibition is another indicator of cytokine-mediated induction of Activin A signaling. SMAD(2)/3 phosphorylation was counteracted by SB431542, αActA, TAK-1 inhibitor, SB203580 and withaferin A (Figure 4C). Inhibition of AKT phosphorylation was also counteracted by SB431542, αActA, and TAK-1 inhibitor, whereas SB203580 and withaferin A were not as effective (Figure 4C).

**Figure 6:**
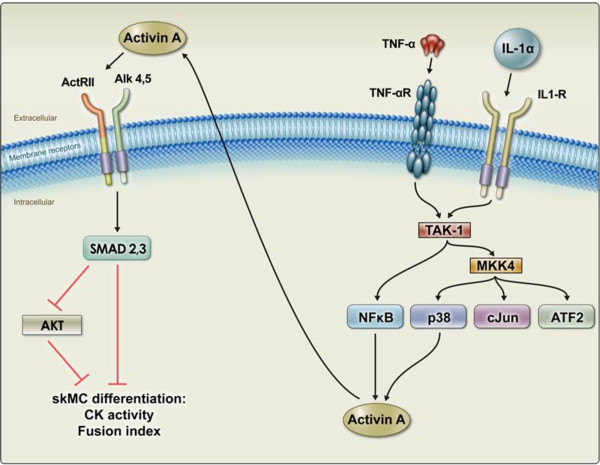
**Pathway model for interleukin (IL)-1**α **and tumor necrosis factor (TNF)-**α**-induced inhibition of human skeletal muscle cell (HuSKMC) differentiation**. IL-1α and TNF-α induced secretion of Activin A in differentiating HuSKMCs cells and this secreted Activin A mediated their effects. Release of Activin A was dependent on **t**ransforming growth factor-β-activated kinase-1/p38/nuclear factor κB pathway activation, but independent of SMAD2/3 signaling. The released Activin A subsequently inhibited differentiation via ALK/SMAD2/3 signaling.

Taken together, these results further support the model that IL-1α and TNF-α cause Activin A secretion via TAK-1/p38/NFκB signaling, and that secreted activin A subsequently signals in an autocrine fashion via ALK/SMAD(2)/3/AKT to inhibit differentiation (Figure 6).

### The transforming growth factor-β-activated kinase-1/p38/Activin A/SMAD3 signaling pathway is upregulated in rat sarcopenia

**Figure 5:**
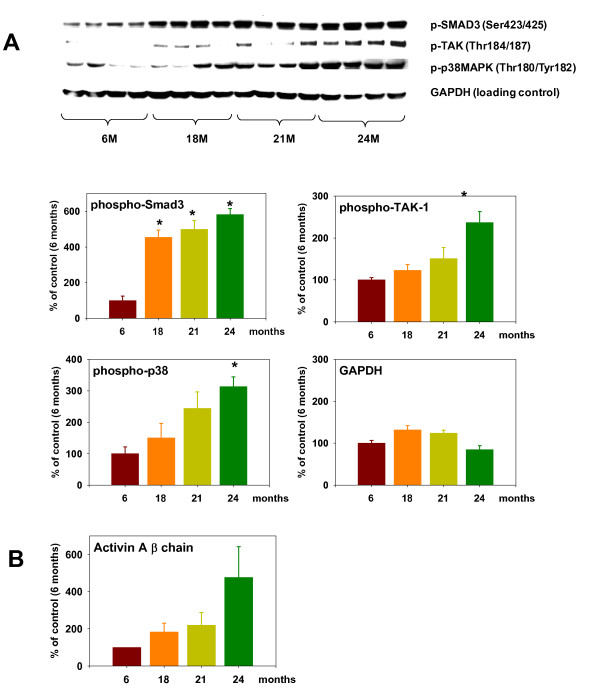
**Transforming growth factor-β-activated kinase (TAK)-1/p38/Activin A/SMAD3 pathway activation in a rat sarcopenia model**. **(A) **Immunoblotting of phospho-SMAD3, phospho-TAK-1, phospho-p38 and GAPDH in samples from rats of different ages. The gastrocnemius muscle was sampled from four rats per age point (6, 18, 12 and 24 months). Shown are representative immunoblots and averaged densitometry data from the blots. **(B) **Analysis of Activin A β-chain mRNA taken from rat gastrocnemius muscle. Data are expressed as percentage of control (first column, 6-month-old rats) and shown as means ± SEM from four individual rats. Differences from control (first column, 6-month-old rats),*P < 0.05.

Sarcopenia (the loss of skeletal muscle associated with advanced age) has been reported to be due in part to impaired muscle-cell differentiation [3]. Therefore, we analyzed muscle samples from rats of different ages, to see if cytokine induction of Activin A and its downstream pathway might contribute to sarcopenia (Figure 5A,B). phospho-SMAD3 significantly increased by up to 5.8-fold between the ages of 6 and 24 months in rat muscle. Similarly, phospho-TAK-1 and phospho-p38 were significantly increased at 24 months, by up to 2.4- and 3.1-fold, respectively. By contrast, GAPDH protein levels were similar at all ages. Expression of Activin A β-chain (Figure 5B) increased with age by up to 4.8-fold, and serum TNF-α levels also increased (data not shown), confirming upregulation of the TNF-α/TAK-1/p38/Activin A/SMAD3 pathway during aging.

## Conclusions

In this study, we found that IL-1α and TNF-α inhibited the differentiation of human myoblasts, and that this inhibition was mediated by the induction of Activin A signaling (Figure 6). The result is an interesting instance of inflammatory cytokine-induced crosstalk, stimulating TGF-β-type signaling. The induction of Activin A secretion downstream of cytokine pathway stimulation suggests a mechanism explaining how cytokines perturb muscle differentiation, because it is well established that TGF-β family members such as myostatin and Activin A can block myoblast differentiation [8,13,42,43]. The inhibition of differentiation by IL-1α and TNF-α was significant, being at least 50%, and as much as 100%, as measured by FI or CK activity.

The stepwise nature of the cytokine pathway activation leading to Activin A secretion and subsequent SMAD activation was shown using both pharmacological and genetic tools. First, we determined, using a direct measurement of Activin A via an ELISA. that there is a 7- to 10-fold induction of Activin A levels in the supernatants of myoblasts after treatment with the inflammatory cytokines IL-1α and TNF-α The selectivity of the induction was shown using a blocking antibody to Activin A, which was able to ablate the induction of SMAD2/3 signaling caused by the cytokines, as opposed to the soluble TGF-β receptor trap, which had no effect.

We found that induction of Activin A resulted in an activation of downstream Activin receptor signaling, via the SMAD2/3 transcription factors, because blockade of Activin A using the neutralizing antibody ablated the IL-1α/TNF-α-induced SMAD2/3 response.

The signaling cascade downstream of TNF-α and IL-1α which resulted in Activin A stimulation, was also determined (Figure 6). We found that TAK-1 activation is required for Activin A secretion, because an inhibitor to TAK-1 blocked the increase in Activin A, and rescued myoblast differentiation. As expected, TAK-1 blockade also inhibited the downstream activation of p38, which is also required for Activin A production, as shown by assessment of SMAD2/3 signaling in cells treated with or without a p38 inhibitor; p38 blockade increased myoblast differentiation. In contrast to the results with p38, inhibition of JNK did not perturb Activin A signaling, establishing the specificity of the TAK-1/p38 pathway. NFκB also contributed to Activin A induction, although p38 inhibition had a much greater effect than NFκB in rescuing differentiation and in blocking SMAD2/3 activation (Figure 6). This pathway was also seen in HuSKMCs for IL-1β, another native pro-inflammatory cytokine acting on IL-1 receptors (data not shown).

There has been some debate in the literature as to whether inflammatory cytokines play a negative or positive role on myoblast differentiation into myotubes. Although it is still possible that there may be a positive role at low concentrations and particular time points, in this study the effect of the cytokines was convincingly anti-differentiation, bolstered by the dramatic induction of an established mechanism for the inhibition of myoblast differentiation. The induction of Activin A by TNF-α and IL-1α may help to explain some of the phenotypes previously reported in aging animals, including humans. There are multiple reports that inflammatory signaling goes up as mammals age, coincident with the onset of sarcopenia [44]. In addition, it has been shown that there is an increase in TGF-β in sarcopenic animals [45]. The data in this study demonstrate that TNF-α/TAK-1/p38/SMAD/Activin A signaling increases coordinately with age, and that this is not a coincidence, but rather cause and effect.

Inflammatory cytokines and the resultant activation of the NFκB pathway have been previously shown to induce skeletal muscle atrophy in differentiated muscle, by activating the E3 ubiquitin ligase, MuRF1 [46]. This study establishes the mechanism for an additional anti-muscle effect of cytokines: the blockade of differentiation by Activin A secretion. The data suggest that treatment of sarcopenia with agents that block the relevant cytokines that activate TAK-1 would not only block the established pro-atrophy effects of NFκB, but would also provide an upstream inhibition of Activin A release, effectively shutting down two pathways that negatively perturb skeletal muscle in sarcopenia and cachexia.

## List of abbreviations

BSA: bovine serum albumin; CK: creatine kinase; DAPI: 4',6-diamidino-2-phenylindole; DM: differentiation medium; DMSO: dimethyl sulfoxide; FCS: fetal calf serum; GM: growth medium; HuSKMCs: human skeletal muscle cells; IGF-1: insulin-like growth factor 1; IL-1α: interleukin 1 alpha; IL-1β: interleukin 1 beta; PBS: phosphate-buffered saline; skBM: skeletal muscle basal medium; SN: supernatant; TAK-1: transforming growth factor-β activated kinase 1; TBS: Tris-buffered salineTGF-β, transforming growth factor beta; TNF-α: tumor necrosis factor alpha.

## Competing interests

All authors are employees of Novartis Institutes for Biomedical Research.

## Authors' contributions

AUT participated in the design of the study, collected the data, analyzed the data, performed the statistical analysis, and wrote and edited the manuscript, AM participated in the design of the study, collected the data, analyzed the data, and edited the manuscript, CJ and JNF collected the data and edited the manuscript, DJG provided advice and guidance as to the study, and wrote and edited the manuscript. All authors read and approved the final manuscript.
